# Comparison of radiation dermatitis between hypofractionated and conventionally fractionated postoperative radiotherapy: objective, longitudinal assessment of skin color

**DOI:** 10.1038/s41598-018-30710-4

**Published:** 2018-08-17

**Authors:** Hideya Yamazaki, Tadashi Takenaka, Norihiro Aibe, Gen Suzuki, Ken Yoshida, Satoaki Nakamura, Koji Masui, Takuya Kimoto, Naomi Sasaki, Tsuyoshi Nishimura, Akihiro Nakashima, Mariko Goto, Kei Yamada

**Affiliations:** 1 Department of Radiology, Graduate School of Medical Science, Kyoto Prefectural University of Medicine, 465 Kajiicho Kawaramachi Hirokoji, Kamigyo-ku, Kyoto 602-8566 Japan; 2 Department of Radiology, Osaka Medical College, 2-7 Daigaku-machi, Takatsuki-City, Osaka 569-8686 Japan

## Abstract

This study aimed to quantitatively compare radiation dermatitis due to hypofractionated (Hypo) and conventionally fractionated (Conv) external-beam radiotherapy in patients who underwent postoperative radiotherapy after breast-conserving surgery. Skin color changes, in terms of L* (brightness, white-black), a* (red-green), and b* (yellow-blue) values, due to external-beam radiotherapy were examined at alternate fractions using an objective method. Twenty-six patients were included in the Hypo group (42.56 Gy/16 fractions) and 46 in the Conv group (50 Gy/25 fractions). Radiotherapy decreased the L* value (darker) and increased the a* value (redder) gradually. These color alterations progressed linearly according to elapsed fractions and were similar between Hypo and Conv per fraction. The Hypo group showed significantly milder alterations in L* and a* values than the Conv group. The maximal dosage was significantly correlated to alterations in L* and a* values. Common Terminology Criteria for Adverse Events v4 assessment did not show a statistically significant difference between the Hypo (Grade 0:1:2 = 2:24:1) and Conv (1:39:6, p = 0.25) groups. The results of our objective analysis revealed that patients undergoing Hypo show milder color alteration than those undergoing Conv and that the maximal dosage is a useful predicator of color alteration.

## Introduction

Breast conservation therapy (BCT) for early-stage breast cancer involves a lumpectomy followed by whole-breast radiotherapy. BCT has become the standard therapy in suitable breast cancer patients, and it can involve either standard conventional fractionation (Conv: e.g., 50 Gy/25 fractions) or accelerated hypofractionated external radiotherapy (Hypo: e.g., 42.56 Gy/16 fractions). Hypo has become a field of interest in radiation oncology. As Hypo is regarded as a good treatment option because it decreases treatment time for patients for whom distance and time are obstacles, several randomized trials have compared various Hypo techniques^1–5^. In general, Hypo does not decrease disease control or worsen long-term cosmetic outcomes^2–5^ and may decrease acute radiation toxicity risk compared to Conv^6^.

However, there is currently no objective universal skin toxicity-rating scale. Hence, there is always a risk of subjective factors interfering with the rating. In most previous studies, subjective methods, such as visual inspection, have been used to determine dermatitis extent^6^, which creates several uncertainties. However, various reliable and reproducible objective assessment tools have recently been introduced^7–12^. Thus, in previous studies, we used an objective measurement to examine radiation dermatitis^12–16^ and reported the usefulness of the objective assessment in estimating the difference between standard external radiotherapy (Conv) and Hypo brachytherapy (accelerated partial breast irradiation) after breast-conserving surgery (BCS)^13^. In this study, we objectively investigated the quantitative differences in radiation dermatitis, in terms of skin color alterations, caused by widely administered Conv and Hypo using a meticulous longitudinal analysis of data obtained for every alternate fraction. We also examined the correlation of the objective measurements with the standard grading of skin toxicity using Common Terminology Criteria for Adverse Events Version 4.0 (CTCAE v4).

## Results

### Longitudinal time course of skin color changes resulting from radiotherapy

**Figure 1:**
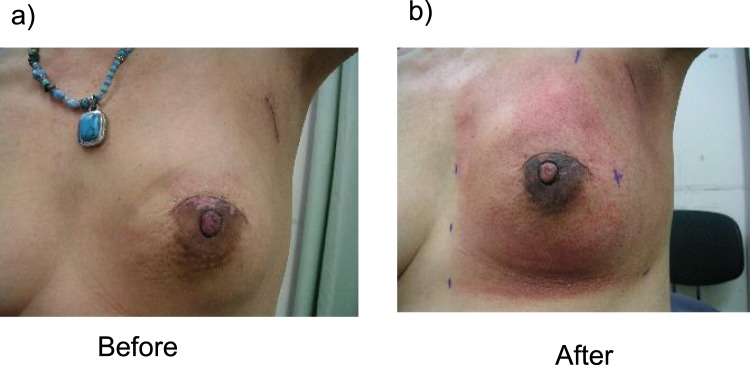
A representative image of grade 2 radiation dermatitis. A 59-year-old female showed grade 2 radiation dermatitis at 50 Gy/25 fractions of postoperative radiotherapy for left breast cancer (scirrhous carcinoma; pT1cN0M0). Her Δa^*^ value was 2.975 (last a^*^ value = 10, initial a^*^ value = 7.025), whereas ΔL^*^ value was −8.625 (initial L^*^ value = 64.125, last L^*^ value = 55.55).

We examined skin color in terms of L*, a*, b* values. The average (±standard deviation) of L* (a*, b*) value was 65.3 ± 2.45 (6.02 ± 1.48, 16.7 ± 1.74) for a treated breast and 67.17 ± 2.70 (5.24 ± 1.25, 16.1 ± 1.67) for an untreated contralateral breast [n = 72, p < 0.0001 (p = 0.0004, p = 0.080)]. This indicated that surgery decreased the L* value (darker) and increased the a* (reddish) value while also possibly decreasing the b* value (yellow). Figure 1 shows a representative image of grade 2 radiation dermatitis.

**Figure 2:**
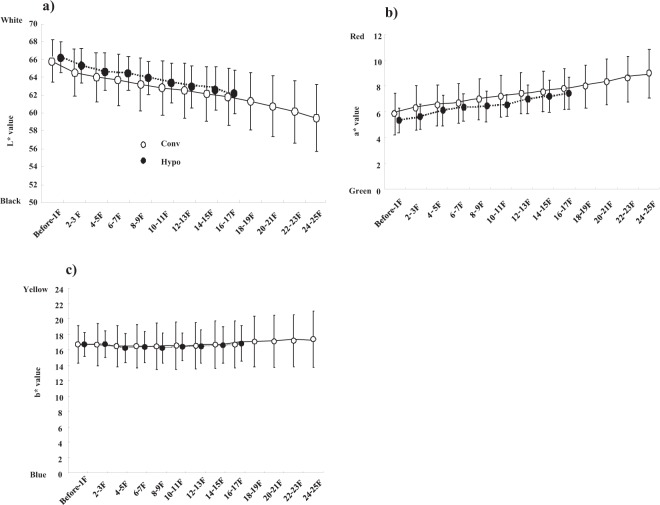
Longitudinal alteration in color values during postoperative radiotherapy. L^*^ and a^*^ values in treated skin changed in a statistically significant manner during the time course (L^*^ value indicates darker, a^*^ indicates redder; L^*^, a^*^ both <0.0001). Conv; conventional radiotherapy, Hypo; hypofractionated radiotherapy. **(a)** Time course of L^*^ values in treated breasts. **(b)** Time course of a^*^ values in treated breasts. **(c)** Time course of b^*^ values in treated breasts. Each symbol represents the average value, with error bars showing one standard deviation (SD).

**Figure 3:**
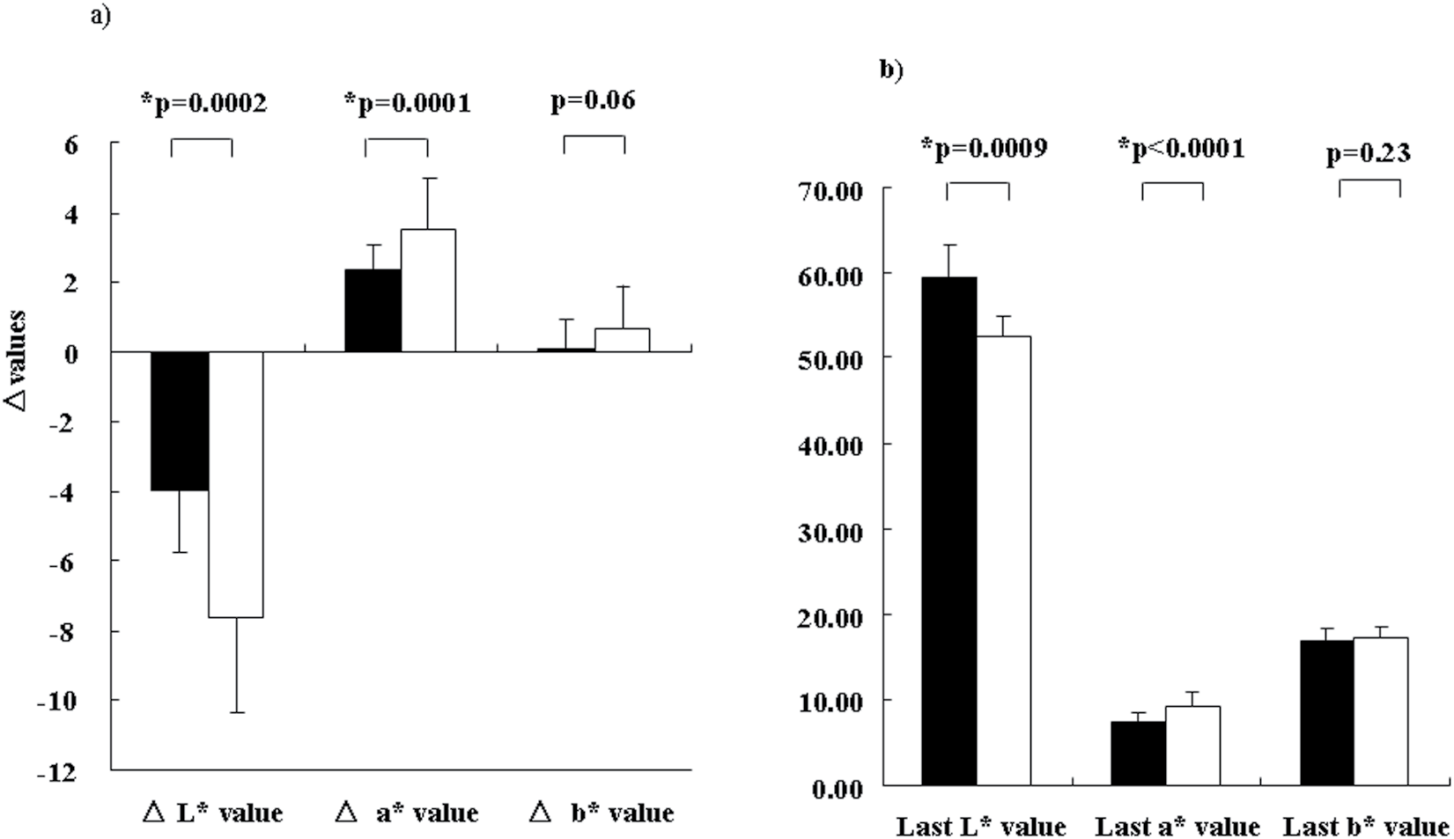
Comparison of L^*^, a^*^ b^*^ alteration between the Hypo and Conv groups. **(a)** Δ values (L^*^, a^*^, b^*^). **(b)** Last values (L^*^, a^*^ b^*^). Closed bar depicts the Hypo groups, and open bar depicts the Conv group.

Figure 2 shows the longitudinal time course of skin color changes in the Hypo and Conv groups. Radiotherapy decreased the L* value and increased the a* value gradually, according to elapsed fractions (or accumulated irradiation dose; both p < 0.0001), but the b* value remained unchanged. Although the difference between the Hypo and Conv groups at each fraction did not differ (Fig. 2), the Hypo group showed a milder alteration in the L* (Δ L* value = −3.95 ± 1.81) and a* (Δ a value = 2.32 ± 0.75) values than the Conv group (Δ L* value: −7.59 ± 2.75, p = 0.0002; Δ a* value: 3.54 ± 1.43, p = 0.0001; Fig. 3). The L* and a* values at the last fractions were also significantly lower and higher, respectively, in the Conv group than in the Hypo group (Fig. 3). The alteration in the L* and a* values was linear and well correlated to the number of fractions. The contralateral breasts showed no significant changes in the skin color analysis.

### Comparison of CTCAE v4 and color analysis

**Figure 4:**
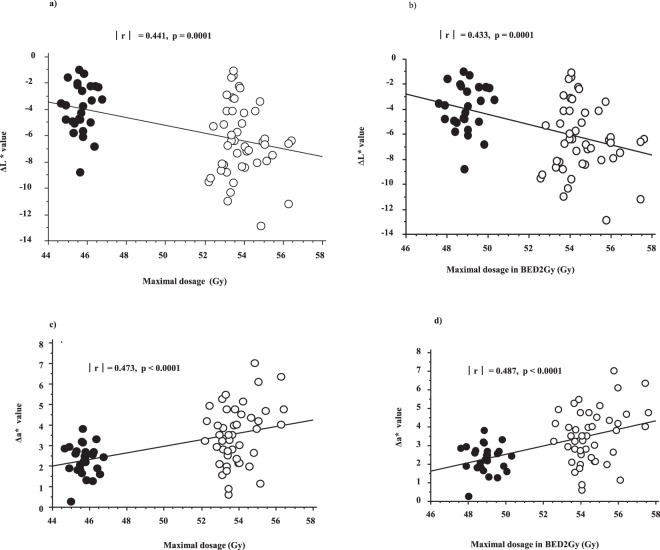
Correlations between skin color alteration and maximal dosage. **(a)** Maximal dosage and ΔL^*^ value. **(b)** Maximal dosage in BED2Gy and ΔL^*^ value. **(c)** Maximal dosage and Δa^*^ value. **(d)** Maximal dosage in BED2Gy and Δa^*^ value. Closed circle depicts the Hypo group, and open circle depicts the Conv group.

We also investigated whether CTCAE v4 for radiotherapy-related skin toxicity correlates with objective measurements of the skin (Table 2). CTCAE v4 assessment classified all cases of dermatitis caused by radiotherapy as grades ≤2, indicating no statistically significant difference between the Hypo (Grade 0:1:2 = 2:24:1) and Conv (1:39:6, p = 0.25) groups. The last L* value showed statistically significant correlations to CTCAE grading (p = 0.01). Although CTCAE grading of dermatitis (Table 2) showed no significant correlation to maximal dosage (with or without correction to BED2Gy), significant intermediate correlations were observed between maximal dosage (with or without BED2Gy correction) and Δ L* and Δ a* values (Fig. 4).

**Table 1:** Characteristics and treatment factors of patients.

Variables	Strata	Hypofractionated RT n=26		Conventional RT n = 46		p-value
		No. or Median (range)	(%)	No. or Median (range)	(%)	
Age		51 (39–75)		54 (35–76)		0.61
Primary site	Right	12	(43%)	19	(40%)	0.87
	Left	14	(50%)	27	(57%)	
Histology	IDC	19	(70%)	31	(67%)	0.72
	DCIS	6	(22%)	11	(24%)	
	Other	1	(4%)	4	(9%)	
pT category	is	6	(22%)	11	(24%)	0.68
	1	16	(59%)	22	(48%)	
	2	4	(15%)	11	(24%)	
	3	0	(0%)	1	(2%)	
	4	0	(0%)	1	(2%)	
pN category	0	24	(89%)	35	(76%)	0.16
	1-	2	(7%)	11	(24%)	
Chemotherapy	Yes	7	(27%)	15	(33%)	0.61
	No	19	(73%)	31	(67%)	
Hormonal therapy	Yes	9	(35%)	13	(28%)	0.57
	No	17	(65%)	33	(72%)	
Examined fraction	Even fraction 2nd, 4th, 6th.	9	(35%)	13	(28%)	0.76
	Odds fraction 1st, 3rd, 5th	17	(65%)	33	(72%)	
Maximal dose	(Gy)	46.4 ± 2.82		53.9 ± 1.7		**<0.0001**

IDC: invasive ductal carcinoma, DCIS; ductal carcinoma *in situ*.

**Table 2:** Parameters according to CTCAE 4.0 grade.

Variables		Grade 0 (n = 3) Average ± SD		Grade 1 (n = 62) Average ± SD		Grade 2 (n = 7) Average ± SD		p-value
Schedule	Hypo	2	(67%)	23	(37%)	1	(14%)	0.25
	Conv	1	(33%)	39	(63%)	6	(86%)	
Last L^*^ value		64.4 ± 2.1		60.7 ± 3.4		57.1 ± 2.8		**0.01**
Δ L^*^ value		−3.9 ± 2.3		−5.3 ± 2.7		−7.2 ± 2.5		0.14
Last a^*^ value		7.7 ± 1.7		8.5 ± 1.8		9.4 ± 1.2		0.19
Δ a^*^ value		2.8 ± 1.1		3.1 ± 1.4		3.6 ± 1.3		0.47
Last b^*^ value		15.8 ± 0.8		17.3 ± 1.4		16.4 ± 0.6		0.07
Δ b^*^ value		0.1 ± 0.6		0.5 ± 1.1		0.7 ± 1.1		0.56
Maximal dosage	(Gy)	49.4 ± 5.9		50.8 ± 4.0		52.6 ± 3.3		0.78
Maximal dosage in BED2Gy	(Gy)	50.0 ± 6.4		51.3 ± 3.6		52.9 ± 3.2		0.44

Bold value indicates statistically significant difference.

CTCAE v4 = Common Terminology Criteria for Adverse Events Version 4.0.

## Discussion

Over the past two decades, the concept of Hypo has created a new perspective on breast radiotherapy^1^. Hypo is now established as an additional standard postoperative radiotherapy to BCT and is covered in Japan’s health insurance system because women who are busy working or caring for infants/elderly relatives may require a shorter treatment time. Hypo is associated with lower rates of acute toxic effects and lower levels of fatigue than Conv^5^. Reportedly, the incidence of acute dermatitis decreased significantly by Hypo compared with that by Conv (36% vs. 69%, respectively; p < 0.001), especially that of ≥grade-2 dermatitis (47% vs. 78%, respectively; p < 0.001)^5^. In our study, grade-2 radiation dermatitis was found in 1/26 patients (4%) in the Hypo group and 6/46 (13%) in the Conv group; these values are lower than those reported previously^5^. This could be, in part, because Japanese women tend to have smaller breasts than Caucasian women, which may have decreased the irradiated volume and toxicity. We also made efforts to decrease the maximum dose. In other words, we used a wedge filter and a field-in-field technique to decrease the maximum dose up to 110% of the prescribed dose as much as possible.

Data form the objective analyses of radiation dermatitis is limited because it is considered to be a minor complication, especially after using high-energy Linac systems. However, this complication should not be neglected because it can be objectively evaluated. In the early days of radiotherapy, radiation dermatitis was recognized as an important indicator for the objective estimation of the effect of radiotherapy, i.e., an “erythema dose” in the 1910s was approximated at 5–6 Gy of a radiation dose and was used as a unit of radiation dosage. Biological indicators are an important aspect of radiation oncology and cannot be replaced by physical indicators. To date, an objective and quantitative method for assessing skin toxicity is not available.

Thus, in this study, an objective and quantitative method for assessing radiation dermatitis due to radiotherapy. First, we evaluated skin color alterations after radiotherapy by examining skin colors before treatment, just after treatment, 1 month after treatment, 6 months after treatment, and 12 months after treatment. We found that the L* value alteration peaked just after or 1 month after treatment, whereas a* value peaked just after treatment (50 Gy/25 fractions ± boost 10 Gy/5 fractions)^12^. Thus, the L* value showed a more delayed recovery than the a* value, indicating that the black color change remained longer than the reddish color change caused by radiotherapy. Next, we explored the role of phototype. Suntan type (darkened skin after 1-h stay at the beach during the summer season) showed higher pigmentation than sunburn type (reddish skin after 1-h stay at the beach during the summer season). In addition, the constitutive skin color category (very light to black) did not affect the severity of radiation dermatitis^14^. Thirdly, higher body weight (or body mass index) predicted a greater reddish change^16^. The quantitative method used in the present study revealed less color alterations in the Hypo group than in the Conv group.

Our research is the first to objectively compare radiation dermatitis caused by Hypo and Conv. We aimed to study how skin color alteration progresses based on the variations in the progression of skin color alterations, e.g., a sigmoid curve, linear curve, or step-by-step change. We found that skin changes were nearly linear and gradual progression during radiotherapy. Therefore, our findings indicate that it is possible to predict the final skin color alteration value (L* or a* value) at an arbitrary and appropriate time point by calculating the elapsed fractions or total prescribed dosage if a baseline value is recorded before radiotherapy. Those earlier prediction of future color alteration will fruitful for patients care.

The current assessment system for skin toxicity, i.e., CTCAE v4, assumes a correlation between the clinician-assessed scoring criteria and skin color measurements because biophysical parameters are expected to be associated with visible changes in the skin (erythema and pigmentation) and therefore might directly reflect the clinician’s assessment. Yoshikawa *et al*. reported a wide variation among clinician-assessed scoring for radiation dermatitis if CTCAE v4 is used, which warrants the development of more objective methods^6^. Thus, we attempted to revive the role of biological indicators by evaluating them using objective and quantitative methods^12–16^. However, Mamm *et al*. concluded that replacing the common CTCAE scoring by objective methods for classifying acute radiation toxicity is not necessary because the objective spectrophotometric measurements in their study were found to correlate well with the subjective CTCAE scores^7^.

Notably, in our setting, the same degree of color changes (blackening and reddening) appeared at similar elapsed fractions both in the Hypo and Conv groups. Unexpectedly, the degree of alteration per fraction between 2 Gy and 2.66 Gy was almost identical between the two groups. Initially, we hypothesized that the Hypo schedule would produce more color alteration than the Conv schedule because of a higher accumulated prescribed dosage for each fraction of Hypo. However, the two schedules showed similar alterations at the same fractions. One possible explanation is that a wide shoulder existed in the dose–response curve among those dose ranges (2–2.66 Gy) or that the difference was smaller than our system could detect. We believe that this aspect requires further exploration.

The use of a prescribed dose of 42.56 Gy in 16 fractions was associated with an excellent outcome^2^ when the prescribed dose in 2-Gy equivalents was 44.9 Gy (BED 2-Gy equivalents in α/β = 10 for skin toxicity), 47.2 Gy (α/β = 4 for breast cancer; 1), and 48.2 Gy (α/β = 3 for late reaction). We performed a correlation analysis between maximal dosage and color change and found statistically significant relationships between maximal dosage (with or without BED2Gy correction) and Δ (a* and L*) values. We confirmed that the dosimetric indicator correlated to color alterations. Although it did not reach a statistically significant level, maximal dosage could be correlated to skin toxicity grading in CTCAE v4 (Table 2) and may have a clinical significance if a larger population could be examined.

Several influential factors have been reported for radiation dermatitis, namely large breast volume, lower radiotherapy energy, wider area irradiation and boost irradiation, and an absence of skin care elevated the severity of radiation dermatitis^17–21^. Among these factors, the inhomogeneity of radiation dose distribution was found to cause severe radiation dermatitis. With conventional breast radiotherapy, a portion of the breast tissue receives 110% of the prescribed dose and occasionally up to 120%^21^. Chen *et al*. reported that receiving .110% of a prescribed dose is an important predicator of radiation dermatitis^20^. Therefore, it was reasonable to decrease the maximum dosage up to 110% of the prescribed dosage as much as possible to prevent severe dermatitis.

Three color models, namely HSB/HSL, RGB, and CIE L*a*b*, can be converted to each other through calculations. We chose the CIE L*a*b* model because it has been authorized by the International Commission on Illumination [the Commission Internationale de l´Eclairage (CIE)] and several commercially available machines support this color model. Therefore, we could conveniently use the available machines in our clinical study if we chose the CIE L*a*b* model.

Skin color was mostly determined by the components of melanin and hemoglobin (including blood flow). Melanin strongly affects the radiosensitivity of the skin. Because the L*a*b* value correlates to the melanin content, several attempts have been made to estimate correct melanin/erythema contents separately from the L*a*b* values. However, this estimation is difficult, and no standard methods are available at present. Therefore, investigating the role of melanin was beyond the scope of this study.

Our study has several other limitations. First, this was a preliminary study with a small number of patients. We might confirm the current results in a future trial with a larger number of patients and may establish a qualitative estimation system for both the L* and a* values and the maximal dosage. Second, our data did not contain the subjective evaluation of symptoms by the patients, which would be important because several studies have found significant differences between patients’ and clinicians’ evaluations when assessing toxicities following radiotherapy or chemotherapy^22–24^. Lastly, our study did not analyze maximal color alteration, which could occur even after the completion of radiotherapy. Drost *et al*. reported that radiation dermatitis peaks approximately 2 weeks after the completion of radiotherapy (50 Gy/25 fractions or 42.56 Gy/16 fractions)^25^. However, it is difficult to have patients visit the hospital daily after completing their treatment, and we hypothesize that the maximal color change is correlated with the delta value.

In conclusion, our study results indicate that an objective analysis can quantify the less-invasive nature of the Hypo schedule and that maximal dosage would be a useful predicator of skin color alteration.

## Methods and Materials

### Patient characteristics and treatment

Between January 2011 and December 2016, 72 patients received postoperative radiotherapy at the Department of Radiology at Kyoto Prefectural University of Medicine. All patients who underwent BCT were conventionally treated with a tangential-field 6-MV photon beam using Linac. The median age of the patients was 44 (range: 38–68) years. Inclusion criteria were invasive or noninvasive ductal or invasive lobular carcinoma, age < 80 years, and an Eastern Cooperative Oncology Group performance status of 0–2. All patients had histologically proven breast cancer. Table 1 presents the patient characteristics. We compared 26 patients in the Hypo group and 46 patients in the Conv group; the patients were allowed to select their preferred schedules. There was no significant difference in the background characteristics between the two groups (Table 1). The Hypo group was scheduled to receive 42.56 Gy in 16 fractions, and the Conv group was scheduled to receive 50 Gy in 25 fractions. The details of radiotherapy have been described elsewhere^12,13^. In brief, the radiation dose was normalized to a point in the midplane of the breast (the ICRU reference point). Images were acquired on a CT-scanner (Aquilion, Toshiba medical, Tokyo, Japan) from the mid-neck to the mid abdomen using 3-mm slices. The CT data were transferred to a commercial treatment planning system (Xio, Electa Medical Systems Inc., Stockholm, Sweden) where planning and maximal dosage in the irradiated area were calculated (global max). To correct the difference of fractionation, maximal dosage in BED2Gy (BED 2-Gy equivalents in α/β = 10 for skin toxicity) was also calculated according to the following equation: BED2 Gy = (number of fractions) × (fraction doses) × [(α/β + fraction doses)/(α/β + 2)]. To obtain a homogenous dose distribution, we used a wedge filter and/or a field-in-field technique so that the maximal dosage would decrease to <110% of the prescribed dosage^17^. Furthermore, we used two additional small sub-beams to achieve a uniform dose distribution throughout the target volume. An additional booster dose of 10 Gy/5 fractions (n = 18) for the Conv group and 10.64 Gy/4 fractions for the Hypo group (n = 11) using 4–10-MeV electron beams was administered to patients with a positive surgical margin of ≤5 mm. Booster doses were not included in color assessment for this analysis.

### Other treatments

Twenty-two patients received chemotherapy (neoadjuvant chemotherapy and/or adjuvant chemotherapy—seven in the Hypo group and 15 in the Convo group) (Table 1). Hormone therapy was administered to nine patients in the Hypo group and 13 patients in the Conv group.

### Skin color alteration

We determined breast skin color at room temperature and under room light with a Color Reader CR-13 (Konica Minolta, Tokyo, Japan). To eliminate skin changes caused by the surgical procedures, we measured breast skin that was a sufficient distance from the surgical wound (≥2 cm) and as flat as possible. In total, 4319 measurements were taken. Skin color was determined in terms of three-dimensional CIE-L*a*b* space approved by the CIE. The L* axis (from 0-black to 100-white) represents the luminance of the sample as it is perceived by the human eye, the b* axis represents the complementary yellow (>0)/blue (<0) color components, and the a* axis represents the complementary red (>0)/green (<0) color components. In terms of skin color, an erythema will make the skin darker and redder, resulting in a reduction in the L* value and an increase in the a* value. We measured four quadrants, A (upper inner), B (lower inner), C (upper outer), and D (lower outer) in irradiated breasts and nonirradiated breasts for control. We investigated the respective time courses of color changes. The first measurements were performed before radiotherapy, and measurements were made at every alternate fraction during radiotherapy (odd group 1^st^, 3^rd^, 5^th^ fraction, etc. or even group 2^nd^, 4^th^, 6^th^ fraction, etc.). The last examination was performed in the 24^th^ (even group) or 25^th^ (odd group) fraction in the Conv group and in the 15^th^ (odd group) or 16^th^ (even group) fractions of the Hypo group. The same person conducted the color assessments on a single patient. We excluded the alteration caused by boost irradiation to simplify the analysis. Contralateral breasts were also assessed once a week as a control. An average of four quadrants was used for the value of each time point^13^.

Simultaneously, we used CTCAE v4, which has become the standard for evaluating radiotherapy-related skin toxicity. An independent observer provided a blind assessment of skin toxicity using the CTCAE v4 colorimeter. ΔL*, a*, and b* was calculated by subtracting the previous value (before radiotherapy) from the last examined value.

All patients were enrolled in the study after obtaining their informed consent prior to radiotherapy; the study was performed according to the guidelines and protocol approved by the intra-institutional ethics committee (IRB) of Kyoto Prefectural University of Medicine (assigned number: RBMR-c-803-2). The participants provided written consent to have their images published in an open-access, online journal.

### Statistical analysis

All statistical analyses were performed with a Statview-v5.0 software program. Student’s *t*-tests were used for normally distributed data and the Mann–Whitney *U*-test (Kruskal–Wallis test for multiple data sets) for skewed data. To analyze the correlation coefficients |r|, we defined p < 0.05 if |r| ≥ 0.2 (0.4 ≥ |r| > 0.2, weak correlation; 0.7 ≥ |r| > 0.4, intermediate correlation; |r| > 0.7, strong correlation). Percentages were analyzed using the chi-square test, and a p value of <0.05 was considered as statistically significant.
